# A Case of Significant Improvement of Heart Failure With Reduced Ejection Fraction With a Small Dose of Candesartan in a Hemodialysis Patient With Hypertensive Heart Disease and Nephrosclerosis

**DOI:** 10.7759/cureus.45062

**Published:** 2023-09-11

**Authors:** Haruhito Harada, Yoshiteru Higa, Daisuke Wakasugi, Yoshifumi Wada

**Affiliations:** 1 Cardiovascular Medicine, Wada Heart and Kidney Clinic, Tosu, JPN; 2 Vascular Surgery, Wada Heart and Kidney Clinic, Tosu, JPN; 3 Internal Medicine, Wada Heart and Kidney Clinic, Tosu, JPN; 4 Nephrology, Wada Heart and Kidney Clinic, Tosu, JPN

**Keywords:** heart failure with reduced ejection fraction, nephrosclerosis, hypertensive heart disease, hemodialysis, diastolic hypertension, candesartan

## Abstract

Hypertension induces vascular damage followed by organ damage, including heart failure in hypertensive heart disease (HHD) and nephrosclerosis (the resultant renal pathologic change from long-standing hypertension affecting renal vascular supply), ultimately causing renal failure. Renin-angiotensin-aldosterone system (RAAS) inhibitors are well known as effective drugs for the treatment of hypertension and the anti-remodeling of affected organs. A 52-year-old male was evaluated. Right atrophic kidney and proteinuria were noted in his high school years; however, he had no symptoms for about 35 years. He had pollakiuria in November and oliguria and leg edema in December 2020. The edema deteriorated rapidly, and general fatigue and orthopnea emerged in January 2021. Anasarca, hypertension (198/151 mmHg), tachycardia (115/minute), and hypoxemia (oxygen saturation {SpO_2_} of 93%) were observed on admission. A bilateral pleural effusion and pulmonary congestion were found on a chest X-ray (CXR) examination. An echocardiogram showed a 22% left ventricle ejection fraction (LVEF). Blood urea nitrogen (BUN) and serum creatinine concentrations were 70 mg/dL and 6.05 mg/dL, respectively. He was diagnosed with nephrosclerosis and HHD-induced cardiac exhaustion. Hemodialysis was started in April 2021. Even though the dry weight was decreased by draining water, cardiomegaly (cardiothoracic ratio {CTR}: 60%), low LVEF (20%-30%), and hypertension, especially diastolic hypertension (140-150/100-120 mmHg), were sustained. After 2 mg of candesartan was added in November 2021, the cardiomegaly, blood pressure (BP), and LVEF were rapidly ameliorated. The CTR and LVEF recovered to 48.5% and 60%, respectively, in April 2022. Statistical analyses showed that the independent factors for CTR were the mean monthly diastolic BP (standard partial regression coefficient {$\beta$}: 0.9058, p<0.0001) and candesartan ($\beta$: -0.7389, p=0.0011) in vital signs and prescribed drugs, respectively. We experienced a case of a significant effect of candesartan treatment against heart failure with reduced ejection fraction (HFrEF) caused by HHD in a hemodialysis patient with nephrosclerosis. Statistical analyses suggested that the improvement of HFrEF resistant to fluid removal by hemodialysis was presumably due to a decrease in diastolic BP caused by a small dose of candesartan.

## Introduction

Hypertension induces vascular damage and stimulates arterial stiffening [1,2]. Arterial stiffening also deteriorates hypertension and ultimately results in the vicious cycle of hypertension and vascular damage [2]. Vascular damage due to hypertension induces various types of organ damage, including stroke, cardiovascular disease, renal dysfunction, and retinal damage [3]. Sustained high arterial pressure increases the afterload of the heart and induces cardiac exhaustion from hypertensive heart disease (HHD), resulting in heart failure [4,5]. The kidney is also an organ easily affected by hypertension [6]. As the kidney is a vascular complex originating from functional small vessels and arterioles, damage to small vessels and arterioles caused by continuous hypertension results in renal dysfunction, i.e., nephrosclerosis [7].

In the reports of the Japanese Society for Dialysis Therapy [8], nephrosclerosis has been the second leading cause of dialysis induction in patients, following diabetic nephropathy, in Japan since 2019, and the incidence rate continuously increases in contrast to diabetic nephropathy. Approximately 20% of mortality in a dialysis-inducing year is caused by heart failure, second to the rate of mortality for infectious disease. The leading cause of mortality in chronic dialysis patients has been heart failure since 1983.

It is important to break the vicious cycle of hypertension and organ damage, including heart failure and renal failure. Renin-angiotensin-aldosterone system (RAAS) inhibitors, including angiotensin II AT-1 receptor blockers (ARBs), are known to have not only antihypertensive effects but also anti-remodeling and target organ protection effects [9]. Ottosson et al. report that steady-state plasma concentrations of candesartan in hemodialysis patients are approximately twice in those subjects with normal renal function, and with the careful monitoring of blood pressure (BP), candesartan can be titrated from 4 mg to 16 mg once daily in hemodialysis patients [10]. And Takahashi et al. reported that candesartan significantly reduced cardiovascular events and improved prognosis in chronic maintenance hemodialysis patients [11]. We experienced the drastic improvement of heart failure with reduced ejection fraction (HFrEF) with a small dose of candesartan in a hemodialysis patient with HHD and nephrosclerosis.

## Case presentation

A 52-year-old male was admitted to our clinic with chief complaints of anasarca, shortness of breath, general fatigue, and nocturnal orthopnea.

Present illness

Right atrophic kidney and proteinuria were noted in his high school years; however, he had no symptoms for about 35 years. He had pollakiuria in November and oliguria and leg edema in December 2020. The edema deteriorated rapidly, and shortness of breath, general fatigue, and nocturnal orthopnea emerged in January 2021.

Family history

His elder brother was on hemodialysis.

Clinical findings on admission

The patient’s consciousness was clear, his BP was 198/151 mmHg, and his pulse was consistently 115/minute. Oxygen saturation (SpO_2_) was 93% on room air. Auscultatory findings revealed a fourth-tone gallop rhythm in heart sounds and coarse crackles in respiratory sounds.

In the renal function test, blood urea nitrogen (BUN) and serum creatinine concentrations were 70 mg/dL and 6.05 mg/dL, respectively. Twenty-four-hour creatinine clearance was 11 mL/minute. The intact parathyroid hormone concentration was 253 pg/mL. An elevated white blood cell count (13680/$\mu$L; differential was 95% neutrophils, 3% lymphocytes, and 2% monocytes) and serum C-reactive protein concentration (5.88 mg/dL) showed the existence of acute inflammation. In the screening of nephritis, secondary hypertension, and secondary myocardial disease, elevated values were found in brain natriuretic peptide (BNP) (1760 pg/mL), plasma renin concentration (78.2 pg/mL), plasma aldosterone concentration (516.6 pg/mL), and serum amyloid concentration (96.9 $\mu$g/mL). Abnormal findings were not found in thyroid function, urinary Bence-Jones protein, myeloperoxidase-anti-neutrophil cytoplasmic antibody, proteinase 3-anti-neutrophil cytoplasmic antibody, antinuclear antibody, anti-glomerular basement membrane antibody, and $\alpha$-galactosidase activity. Serum sodium and potassium concentrations were 139 mEq/L and 3.5 mEq/L, respectively.

Cardiomegaly, bilateral pleural effusion, and pulmonary congestion were found on the chest X-ray (CXR) examination. High voltage and left atrial overload were found on the electrocardiogram (ECG). The echocardiogram showed left ventricular dilation (66 mm of left ventricular diastolic diameter) and 22% left ventricle ejection fraction (LVEF). A right renal atrophy (73×38 mm) and bilateral unclear central echo complex were found on the renal echogram. No obvious stenosis was found in the bilateral renal arteries. Special image examinations were performed at Kurume University Hospital. Renal scintigram with technetium (Tc)-99m diethylene-triamine-pentaacetate (DTPA) revealed a dysfunction pattern in the left kidney and a hypofunction pattern in the right kidney. No abnormal findings were found in the computed tomography of the chest and abdomen, bone scintigram, nerve conduction velocity, and coronary angiography. Amyloid protein was not found in either cardiac or fat tissues.

Based on the above findings, we refused secondary hypertension, nephritis, and myocardial diseases and diagnosed essential hypertension, HFrEF by HHD-induced cardiac exhaustion, and renal failure due to nephrosclerosis and right renal atrophy.

Data collection and statistical analysis

Physical and laboratory data were collected from the patient’s medical record. The analysis of variance (ANOVA) and Student’s t-test were performed to analyze blood pressure (BP) changes. The Mann-Whitney U test was performed to compare the median cardiothoracic ratio (CTR) before and after treatment with candesartan. Nonnumerical data on drug use were changed to dummy numbers and then used for association analyses. Single and stepwise multivariate regression analyses and binomial logistic regression analysis were performed in association studies. All statistical analyses in this report were performed by using BellCurve (Social Survey Research Information Co., Ltd., Tokyo, Japan) for Excel software (Microsoft® Corp., Redmond, WA). Statistical significance was indicated by a p-value of less than 0.05.

Clinical progress

**Figure 1:**
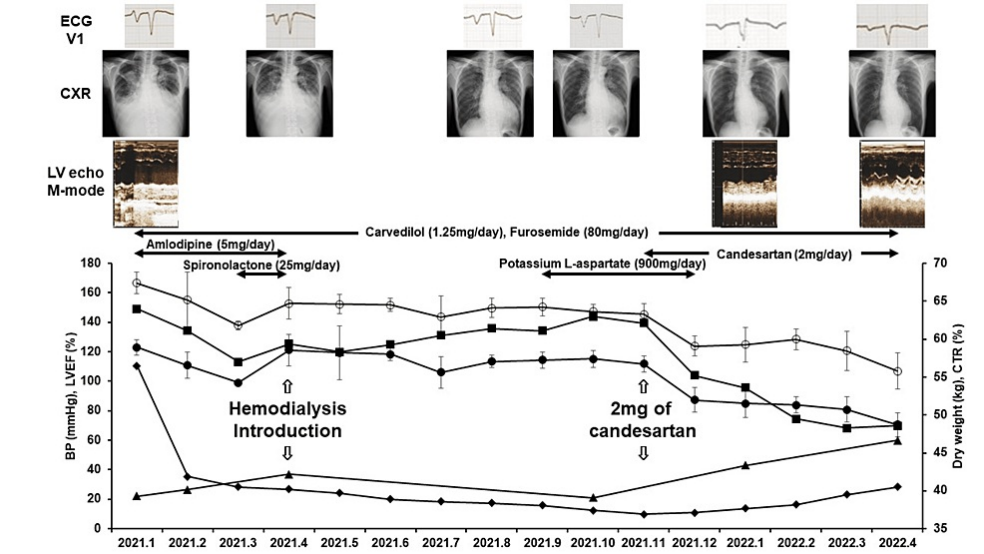
Therapy, clinical progress, and point changes on electrocardiogram (ECG), chest X-ray (CXR), and echocardiogram The negative phase of the P wave in lead V1 of the ECG, indicating left atrial overload, improved after candesartan administration. After starting hemodialysis, the pleural effusion decreased on CXR, but the CTR sustained above 60%. After candesartan administration, the CTR improved to 53.8% at two months and 48.5% in normal range at five months. The LVEF on M-mode echocardiogram, which was 22% on admission, increased to 43% at two months and 60% at five months after candesartan administration. Open and solid circles represent mean monthly systolic blood pressure (BP) and diastolic BP, respectively. Squares represent CTR. Diamonds represent dry weight. Triangles represent LVEF. ANOVA showed statistically significant changes in systolic BP (p<0.0001) and diastolic BP (p<0.0001) LV, left ventricle; LVEF, left ventricle ejection fraction; CTR, cardiothoracic ratio; ANOVA, analysis of variance

To decrease hypertension, we first prescribed 5 mg of amlodipine, but the effect lasted only two months. Hemodialysis introduction in April 2021 was effective for improving anasarca, pleural effusion, and nocturnal orthopnea. However, even though the dry weight was decreased by draining water during hemodialysis, cardiomegaly (CTR: 60%), low LVEF (20%-30%), and hypertension (140-150/100-120 mmHg), especially diastolic hypertension, were sustained (Figure 1).

After 2 mg of candesartan was added in November 2021, the cardiomegaly, LVEF, and BP had ameliorated significantly (Figure 1 and Table 1).

**Table 1:** ANOVA analysis for the change of mean monthly blood pressure ANOVA: analysis of variance

	F value	p-value
Systolic blood pressure	28.8326	<0.0001
Diastolic blood pressure	41.5862	<0.0001

In a comparison of BP values from before and after candesartan treatment, mean systolic BP decreased significantly from 151 mmHg to 124 mmHg (p<0.0001), and mean diastolic BP decreased significantly from 116 mmHg to 87 mmHg (p<0.0001) (Table 2).

**Table 2:** Comparison of blood pressure by Student’s t-test before and after candesartan prescription

	Before candesartan, n=101	After candesartan, n=79	p-value
Mean systolic blood pressure, mmHg	151±10	124±16	<0.0001
Mean diastolic blood pressure, mmHg	116±11	87±15	<0.0001

The median CTR significantly decreased from 60.8% to 51.6% (p=0.0196). The CTR and LVEF recovered to 48.5% and 60%, respectively, by April 2022 (Table 3).

**Table 3:** Median cardiothoracic ratio comparison by Mann-Whitney U test before and after candesartan prescription

	Before candesartan, n=10	After candesartan, n=6	p-value
Median cardiothoracic ratio, %	60.8	51.6	0.0196

Left atrial overload in electrocardiogram (ECG) also improved, accompanied by CTR and LVEF improvements.

Statistical analyses

To explore the effective factor(s) for HFrEF improvement, we performed association studies. Because a significant correlation was found between CTR and LVEF in single regression analysis (n=6, depending on the number of LVEF examinations; R^2^=0.9603; p=0.0006) (Table 4), CTR (n=16), of which the number of examinations was more than that of LVEF, was used for subsequent analyses as a representative marker of heart failure.

**Table 4:** Single regression analysis for CTR and LVEF B, partial regression coefficient; CI, confidence interval; SE, standard error; R, correlation coefficient; CTR, cardiothoracic ratio; LVEF, left ventricle ejection fraction

	B	SE	95% CI	R	R^2^	p-value
CTR versus LVEF	-0.3906	0.0397	-0.5008 to -0.2803	-0.9799	0.9603	<0.0006
Constant term	71.9278	1.4907	67.7890 to 76.0665	-	-	<0.0001

First, single regression analysis was performed between CTR and vital signs, dialysis-related factors, and prescribed drugs. As shown in Table 5, the mean monthly systolic BP, mean monthly diastolic BP, mean monthly heart rate, candesartan, and potassium L-aspartate were significantly related to CTR.

**Table 5:** Single regression analysis of cardiothoracic ratio

Variables	Correlation coefficient	p-value
Vital signs		
Systolic blood pressure	0.8851	<0.0001
Diastolic blood pressure	0.9058	<0.0001
Heart rate	0.7376	0.0011
Hemodialysis markers		
Hemodialysis	-0.2932	0.2704
Dry weight	0.2697	0.3142
Hemoglobin	0.1591	0.5536
Drugs		
Amlodipine	0.3148	0.2349
Candesartan	-0.7389	0.0011
Febuxostat	0.3148	0.2349
Spironolactone	0.0416	0.8784
Potassium L-aspartate	0.5374	0.0318
Lanthanum carbonate	-0.3706	0.1576
Erythropoiesis-stimulating agent	-0.3706	0.1576
Vitamin D	-0.3706	0.1576
Levocarnitine	-0.3706	0.1576
Furosemide	Not estimated	Not estimated
Carvedilol	Not estimated	Not estimated

Second, we performed stepwise multivariate regression analysis to explore independent factors for CTR in the aforementioned significant variables in each category. The independent factors for CTR were the mean monthly diastolic BP (standard partial regression coefficient {$\beta$}: 0.9058, p<0.0001) and candesartan ($\beta$: -0.7389, p=0.0011) in vital signs and prescribed drugs, respectively (Table 6).

**Table 6:** Stepwise multivariate regression analysis of cardiothoracic ratio in vital signs and drugs, respectively Systolic blood pressure (BP), heart rate, and potassium L-aspartate did not associate at p>0.2 B, regression coefficient; $\beta$, standard partial regression coefficient; CI, confidence interval; SE, standard error

Variables	B	SE	95% CI	$\beta$	p-value
Vital sign					
Diastolic BP	0.2664	0.0333	0.1950 to 0.3379	0.9058	<0.0001
Constant term	30.0879	3.4914	22.5996 to 37.5762	-	<0.0001
Drug					
Candesartan	-7.6267	1.8588	-11.6134 to -3.6399	-0.7389	0.0011
Constant term	60.5100	1.1383	58.0686 to 62.9514	-	<0.0001

Candesartan was significantly associated with mean monthly diastolic BP by binomial logistic regression analysis ($\beta$: -3.0663, p=0.0276) (Table 7).

**Table 7:** Binomial logistic regression analysis of candesartan use on the mean diastolic blood pressure (BP) B, partial regression coefficient; $\beta$, standard partial regression coefficient; CI, confidence interval; SE, standard error

	B	SE	95% CI	$\beta$	p-value
Mean diastolic BP	-0.1805	0.0819	-0.3411 to -0.0199	-3.0663	0.0276
Constant term	17.9631	8.5190	1.2661 to 34.6600	-	0.0350

## Discussion

Hypertension and heart failure easily occur in the patient because the excretion of fluid and salt is impaired in hemodialysis patients. Generally, draining water in hemodialysis and extracorporeal ultrafiltration methods are effective for hypertension and congestive heart failure (CHF) in patients on hemodialysis. This water drainage helps heart failure by reducing the preload to the heart and improves pump efficiency. However, they were not effective in this case, and both hypertension and CHF were sustained. Amlodipine, carvedilol, furosemide, and spironolactone also had no effect on the improvement of heart failure and hypertension. A small dose of candesartan drastically improved hypertension, especially CHF associated with diastolic hypertension. The improving effect of candesartan on heart failure and diastolic hypertension was confirmed by statistical analyses.

RAAS inhibitors are well known to be effective drugs for heart failure by decreasing preload and afterload. RAAS inhibitors are expected to reverse and prevent the remodeling of cardiovascular organs [12,13]. It is recommended that the prescribed dose be maximized as much as possible to improve prognosis [14]. Reverse remodeling was reported to be more likely in younger patients (less than 60 years old), those with a systolic BP of >100 mmHg, and those with lower BNP concentrations [15,16]. While the BNP concentration had a high value of 1760 pg/mL, his age of 52 years was relatively young. The Japanese Society of Hypertension and the Japanese Society of Nephrology recommend that antihypertensive medications, including ARBs, be started at low doses to rule out the risk of hypotension due to fluid drainage during hemodialysis. The recommended dose of candesartan is 2 mg at the initial dose for hemodialysis patients. Fortunately, in the present case, 2 mg of candesartan showed a significant antihypertensive effect and cardiac reverse remodeling.

Decreasing intravascular fluid volume and peripheral vascular resistance is an important strategy to treat diastolic hypertension. To the best of our knowledge, there have been only a few reports concerning diastolic hypertension and CHF and the necessity of treatment for diastolic hypertension, although the type of antihypertensive medication is not mentioned [17,18]. On the other hand, reports from Finland and Japan revealed that no significant difference was found in cardiovascular mortality between patients with isolated diastolic hypertension and subjects with normotension [19,20]. These reports suggested the ambiguity of aggressive treatment for diastolic hypertension. In this case, a significant association was found between decreased diastolic BP and the improvement of CHF. As the patient was middle-aged, vascular elasticity might be maintained in the aorta and large arteries. Conversely, the resistance of peripheral arteries and arterioles might be increased, resulting in diastolic hypertension. We hypothesized the following two reasons for the effectiveness of low-dose candesartan in this case: the sensitivity of vasodilation by blocking the AT-1 receptor and activating the Mas receptor with candesartan was suggested to be much higher in the peripheral arteries and arterioles of this patient [21]. Alternately, AT-2 receptor expression, which works for vasodilation, may have been elevated due to the rapid increase in his BP [22]. Decreasing intravascular fluid volume by draining water in hemodialysis was not significantly effective for an antihypertensive effect and the improvement of CHF in this patient (Figure 1).

We had two points worthy of special consideration. First, we delayed prescribing candesartan because we expected the draining water with hemodialysis to have antihypertensive effects and to improve CHF. In general, RAAS inhibitors, including ARB, should be used from the early stage of CHF treatment. Second, amlodipine at the initial stage of the treatment decreased BP temporarily; however, the BP rose again. If the dose had been increased and the use of amlodipine had been sustained and/or the $\beta$-blocker dose had been increased in combination with draining water with hemodialysis, antihypertensive effects and the improvement of CHF could have been observed.

## Conclusions

We experienced a case of a drastic effect of candesartan against hypertension and HFrEF caused by HHD-induced cardiac exhaustion in a hemodialysis patient with nephrosclerosis. Rapidly, a significant cardiac reverse remodeling was shown after the candesartan prescription. Statistical analyses suggested that the improvement in draining water-resistant HFrEF was presumably due to a decrease in diastolic BP resulting from a decrease in peripheral vascular resistance caused by a small dose of candesartan. Considering that heart failure accounts for 20% of mortality in the year of the induction of hemodialysis in Japan, the significant effect of candesartan in this case may contribute to improved patient prognosis.
